# Comparison of Dexamethasone Versus Methylprednisolone With Bupivacaine in Transversus Abdominis Plane Block for Attenuation of Chronic Postoperative Abdominal Pain

**DOI:** 10.7759/cureus.47243

**Published:** 2023-10-18

**Authors:** Anjali Modak, Amreesh Paul, Vivek Chakole, Neeta Verma

**Affiliations:** 1 Anaesthesiology, Jawaharlal Nehru Medical College, Datta Meghe Institute of Higher Education and Research, Wardha, IND

**Keywords:** bupivacaine, dexamethasone, methyl prednisolone, transversus abdominis plane block, chronic abdominal pain

## Abstract

Background

Opioids, which have well-known adverse effects such as drowsiness paralytic ileus and respiratory depression, were mostly utilised to treat postoperative pain in the past. The increased incidence of side effects has led to a rise in interest in pain management techniques that spare opioids. Persistent abdominal pain following surgery has a major detrimental effect on patients' quality of life. While epidural analgesia is widely regarded as the gold standard to combat the pain that is present post abdominal surgeries, it is not devoid of drawbacks. The transversus abdominis plane (TAP) block has developed as a potentially effective treatment for severe abdominal pain. The TAP block acts on the neuro-fascial plane between the internal oblique and transversus abdominis muscles, which is innervated by spinal nerves from T6 to L1. Studies reveal that the addition of corticosteroids to bupivacaine in TAP blocks provides pain relief and improves the quality of life of the patient.

Aims and objectives

In this study, the effects of bupivacaine and corticosteroids, particularly dexamethasone and methylprednisolone, on chronic abdominal pain following surgery are examined. Assessing the quality of pain relief is the primary objective.

Methodology

Thirty patients who had undergone abdominal surgery and had been having persistent abdominal pain for six to eight months thereafter and had attempted unsuccessfully to treat the pain with alternative pain relief methods participated in the study. They were divided into two groups at random. Dexamethasone and bupivacaine were given to patients in Group D while methylprednisolone and bupivacaine were given to patients in Group M for ultrasonography (USG)-guided bilateral TAP blocks. At various intervals up to 12 weeks after injection, the patient's pain levels were measured using the visual analogue score (VAS), and their quality of life was assessed using the quality-of-life score.

Results

Patients in Group M experienced significantly less pain than those in Group D at the fourth, sixth, and 12th weeks of treatment. Furthermore, in the fourth, sixth, and 12th weeks, patients in Group M reported a superior quality of life in comparison to those in Group D.

Conclusion

Patients with persistent postoperative abdominal pain receiving bupivacaine and methylprednisolone in an ultrasonography-guided TAP block experience more effective and long-lasting pain relief than those who receive bupivacaine and dexamethasone. The quality of life for patients may be enhanced by using corticosteroids to optimise postoperative pain management strategies and lessen the need for opioids, as this study highlights.

## Introduction

Historically, the cornerstone of postoperative pain management has been opioids. However, a trend in postoperative pain management towards opioid-sparing methods has occurred as a result of a greater understanding of its side effects, which include respiratory depression, paralytic ileus, and drowsiness [1]. Specifically, patients may experience significant challenges and anguish for months after surgery due to persistent stomach pain. The most effective way to treat pain following abdominal procedures is to utilise an epidural. However, epidural analgesia is uncomfortable due to side effects and complications [2]. For immediate postoperative abdominal pain, the transversus abdominis plane (TAP) block is a potentially effective treatment. It has several benefits, such as a minimal impact on intraoperative hemodynamics and safety for patients experiencing surgical site infections or coagulopathies. The parietal peritoneum, muscles, and skin are the constituents of the anterior abdominal wall. Sensory nerve fibres that extend from T6 to L1 and come from the anterior rami of spinal neurons innervate these tissues. The nerves that provide sensory innervation are the ilioinguinal and iliohypogastric nerves, which originate from L1, the subcostal nerve, which arises from T12, and the intercostal nerves, which arise from T6 to T11. All of these nerves are located in the fascial plane, which occupies the space between the transversus abdominis and the internal oblique muscle [3]. A commonly used method for reducing pain following abdominal surgery is the TAP block [4]. The nerves in this neurofascial plane are anaesthetized using the TAP, a triangle block through Petit's lumbar triangle [5]. The iliac crest forms the triangle's base, and the latissimus dorsi and external oblique muscle, respectively, indicate the triangle's anterior and posterior boundaries. During a TAP block, local anaesthetics are injected between the internal oblique and the transversus abdominis muscles. TAP block techniques include posterior, subcostal, and lateral approaches [6]. In patients who underwent lower abdominal procedures, the posterior TAP block performed better than the lateral TAP.

The anatomic TAP block at the Petit triangle was illustrated in 2001 by Rafi [5]. Hebbard demonstrated an ultrasonography (USG) guided TAP block in 2007 [7]. Historically, this procedure has served to manage abdominal pain, which is acute, mainly pain following the surgery. The role of this block in chronic pain syndromes needs to be studied. The duration of analgesia can be extended by including a corticosteroid. Limited data on the choice and dose of steroids on time and the quality of pain relief are available. Complications of transversus abdominis include bowel hematoma, transient palsy of the femoral nerve, perforation of the peritoneum, perforation of the diaphragm, vascular injury, local anaesthetic toxicity and liver lacerations [8]. With the ultrasound-guided approach, the aforementioned TAP block concerns could be lessened since it enables real-time viewing of the needle tip and pertinent anatomical structures, increasing the margin of safety [9].

The most commonly used local anaesthetics in TAP block include bupivacaine, ropivacaine, and levobupivacaine. The optimal time for TAP block might be the preoperative period as it could decrease early visual analogue scale (VAS) score and opioid usage compared to the postoperative period. Numerous adjuvants have been utilised in the regional block and other peripheral nerve treatments to prolong and enhance the local anaesthetic effect [10,11]. Vasoconstrictors like adrenaline, opioids like fentanyl, tramadol, agents like liposomal bupivacaine, corticosteroids like dexamethasone, triamcinolone acetonide, and methylprednisolone have been used as adjuvants along with local anaesthetics to perform TAP block. Systemic corticosteroids combined with local anaesthetics for analgesic effects have been widely used in human studies [12,13]. To increase the duration of the block, methylprednisolone and dexamethasone have been used in various human and animal studies. It has been demonstrated that dexamethasone has anti-inflammatory properties [14].

The goal of this study is to increase the effectiveness of the TAP block by investigating the effects of corticosteroids, namely dexamethasone and methylprednisolone, in combination with bupivacaine. This study attempts to provide insight into these corticosteroids' potential to reduce persistent post-operative abdominal discomfort by comparing their effects. The secondary objective is to analyse the effect of these combinations on patients' quality of life and the incidence of issues associated with the block. Understanding the analgesic mechanisms of these corticosteroids, including their effects on potassium channel regulation and anti-inflammatory properties, can help optimise post-operative pain management techniques while lowering the need for opioids.

## Materials and methods

Study design

This prospective, randomized experiment was to evaluate the effectiveness of dexamethasone and methylprednisolone in conjunction with bupivacaine used in bilateral TAP blocks for the treatment of persistent abdominal pain. The study was carried out at the Anaesthesiology Department of Acharya Vinoba Bhave Rural Hospital (associated with Jawaharlal Nehru Medical College), Wardha, Maharastra, India, from December 2019 to May 2020. The Institutional Ethics Committee, Jawaharlal Nehru Medical College, Datta Meghe Institute Of Medical Sciences granted ethical clearance (approval number: DMIMS(DU)/IEC/Dec-2019/8597), and each participating patient provided signed informed consent.

Patient selection

**Figure 1:**
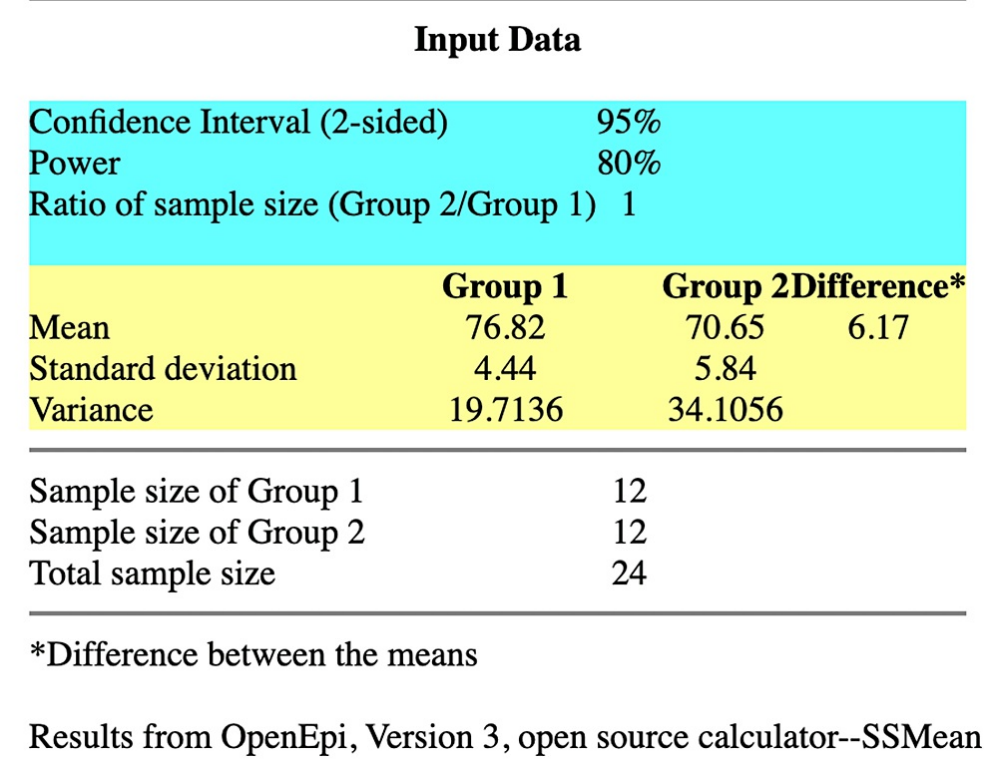
Sample Size Calculation

The trial included patients classified as American Society of Anesthesiologists (ASA) class I and class II, aged between 18 and 60 years, who had been experiencing persistent abdominal pain for a period of six to eight months following abdominal surgery. These patients had previously attempted various forms of chronic pain management and were willing to undergo USG-guided bilateral TAP block. Exclusion criteria encompassed patients with a history of allergic reactions to local anaesthetics, diabetes mellitus, coagulopathy, and hepatic or renal dysfunction. The sample size was calculated using openepi.com (Figure 1) by using means and standard deviation of diastolic blood pressures between the groups according to the study done by Thakur et al. [15]. The sample size required to obtain a statistical significance between both groups was 24. Hence, the study was carried out on 30 patients accounting for any possible dropouts.

Group randomization and data collection

The participants were randomly assigned to two groups (Group D and Group M) of 15 patients each by means of a computer-generated random number table. Demographic information, medical history, and medication usage details were documented for all patients. Vital parameters were recorded, and patients were positioned in the supine position in preparation for the TAP block. An anesthesiologist not involved in patient care, TAP block administration, or data collection prepared the injections for both groups.

Block procedure

To locate the plane between the transversus abdominis and internal oblique muscles, the TAP block was carried out under ultrasound supervision. By using a 3.8-cm 5-10 MHz linear array probe, a single-injection TAP block was administered to each patient. Three millilitres of 1% lignocaine was locally instilled into the location prior to the block. Under ultrasound guidance, a 22-gauge spinal needle was inserted using an in-plane method into the TAP. The plane expanded and became apparent as a hypoechoic zone after the local anaesthetic solution was injected into it. Prior to injection, aspiration was done to rule out arterial puncture. The TAP block on the other side was accomplished using the same technique and medication combination. Group D participants got bilateral TAP block, each side receiving 8 mg of dexamethasone dissolved in 9 cc of 0.5% bupivacaine. Group M participants underwent bilateral TAP block, with 40 mg of injectable methylprednisolone acetate solution in 9 ml of 0.5% bupivacaine administered on each side.

Outcome measures

The primary outcome measure was pain relief, assessed using the VAS for pain at the time of the block and followed up at two, four, six, and 12 weeks post injection during in-person evaluations. The secondary outcome was the assessment of patients' quality of life using the quality-of-life score.

Statistical analysis

While percentages were used to communicate qualitative data, mean values and their related standard deviations were used to describe quantitative data. The student t-test was used to examine quantitative data, and the Chi-square test was used to evaluate qualitative data. Software from Graph Pad Prism version 6.0 (2015; GraphPad Software Inc., San Diego, California, United States) and IBM SPSS Statistics for Windows, Version 20.0 (Released 2011; IBM Corp., Armonk, New York, United States) were used for the statistical analysis, with a significance level of p < 0.05.

## Results

**Figure 2:**
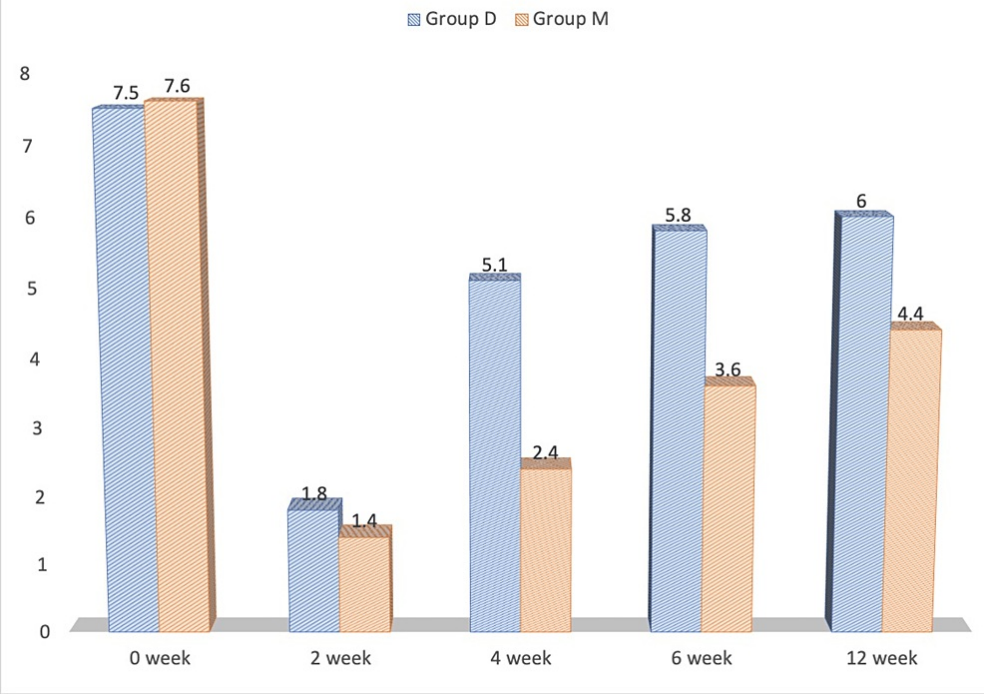
Comparison of VAS score at various time intervals during the study VAS: visual analogue scale

Table 1 shows that the individuals in the two groups were analogous to each other with regard to age, weight, height, and duration of pain (in months). Between the two groups, the patients' mean VAS scores at zero and two weeks were similar. At zero weeks, Group D's mean VAS score was 7.5 ± 0.4, whereas Group M's mean score was 7.6 ± 0.2. After two weeks, Group D's mean VAS score was 1.8 ± 1.0 and Group M's mean score was 1.4 ± 1.2. In the fourth, sixth, and 12th weeks, the mean VAS for group D patients was 5.1 ± 0.8, 5.8 ± 0.8, and 6.0 ± 0.2, respectively. In the fourth, sixth, and 12th weeks of group M, the mean VAS values were 2.4 ± 1.6, 3.6 ± 1.6, and 4.4 ± 1.4, respectively. In the fourth, sixth, and 12th weeks, the mean VAS within group M was considerably lower (p<0.05) than that of group D. Figure 2 shows the comparison of VAS scores.

**Table 1:** Demographics of Group D and Group M

	Group D	Group M	p-value
Age (in years)	45.06±4.48	44.13±5.57	0.67
Weight (in kgs)	64.4±5.36	66.86±5.59	0.162
Height (in cms)	172.27±7.53	173.27±5.85	0.626
Duration of pain (in months)	6.42±0.41	6.62±0.53	0.166

**Figure 3:**
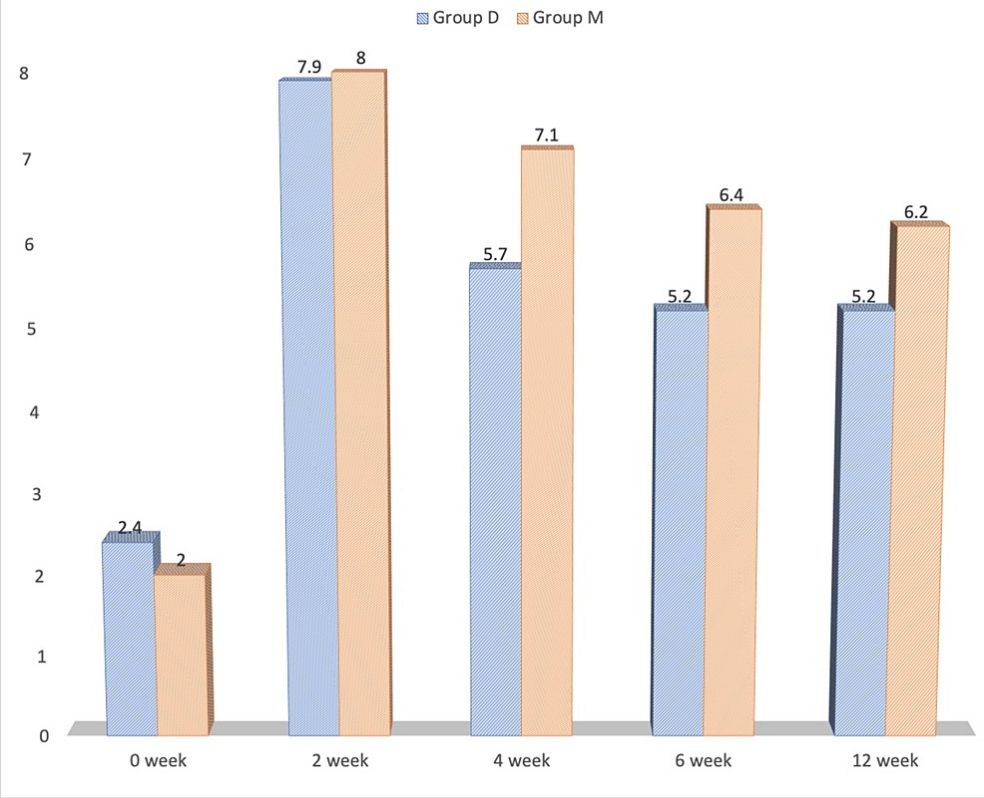
Comparison of quality of life score at various time intervals between the study groups

As shown in Figure 3, the quality-of-life score was compared between study groups at various intervals. The patients' quality-of-life scores at zero and two weeks showed no differences between the two groups. At zero weeks, Group D's quality-of-life score was 2.4 ± 0.2 while Group M's score was 2.0 ± 0.8. After two weeks, Group D's quality-of-life score was 7.9 ± 0.8 while Group M's score was 8.0 ± 0.2. In the fourth, sixth, and 12th week periods, the quality-of-life scores for group D patients were 5.7 ± 1.2, 5.2 ± 1.2, and 5.2 ± 1.2, respectively; in contrast, group M participants' scores were 7.1 ± 0.2, 6.4 ± 0.2, and 6.2 ± 1.2, respectively, at these same intervals. In the fourth, sixth, and 12th weeks, group M's quality-of-life score was considerably higher (p<0.05) than that of group D.

## Discussion

Pain can be defined as an unpleasant, emotional, complex and sensory feeling associated with injury to the tissues [16,17]. The Joint Commission on Accreditation of Healthcare Organizations has recognised pain as the fifth vital sign in addition to blood pressure, respiration, pulse, and temperature [17,18]. Abdominal pain is a many-sided problem which is chronic and challenging. Many factors should be considered for the management of the patient before an ideal mean is selected. The options to treat pain abdomen are mainly opioids and membrane-stabilising agents. Because of factors like work life, social life, and medical contraindications, some patients cannot be on opioids. According to Mallick‐Searle and Fillman, nausea is experienced by 25% of the patients who use opioids for managing pain, 20% experienced vomiting and 23.8% experienced proficient sedation [19]. The other adverse effects include tolerance, hyperalgesia, addiction, hypothalamic-pituitary-adrenal dysregulation, and hypogonadism [10]. Splanchnic nerve block, neuro-modulation, coeliac plexus block and splanchnic radiofrequency ablation have improved visceral pain [20,21]. Limited options are left for patients and providers when these pain management methods are ineffective. To improve patient quality of life, innovative and effective ways of alleviating chronic abdominal pain, besides opioids, are necessary. One such method used is TAP block using bupivacaine with dexamethasone or methylprednisolone as an adjunct [22].

Multiple theories have been postulated as the mechanism of action for the analgesia of steroids. Corticosteroids produce an analgesic effect through immunosuppressive and anti-inflammatory effects. The modulation of nuclear transcription by steroids can result in its analgesic action. Corticosteroids, through their modulation action in potassium channels of excitable cells, can increase the duration of action of local anaesthetics [23]. Some studies have shown that the systemic effects of corticosteroids bring up analgesic effects. 

Dexamethasone is an isomer of betamethasone and a fluorinated analogue of prednisolone. Hydrocortisone is 40 times less effective than dexamethasone [24]. Dexamethasone 0.75 mg provides an anti-inflammatory effect equivalent to 20 mg of cortisol [25]. Due to its water solubility, dexamethasone sodium phosphate is suitable for parenteral administration. When treating specific kinds of cerebral oedema, this corticosteroid is frequently used. Inhibition of inflammatory cells and regulation of the expression of inflammatory mediators are the primary mechanisms by which the anti-inflammatory action is produced. Dexamethasone is a potent drug for preventing and treating post-operative nausea and vomiting. Dexamethasone can be used as a local anaesthetic adjuvant by its action on nociceptor C fibres transmission and neural discharge through its blocking effect and anti-inflammatory effects [26]. Dexamethasone has been found to induce peri-neural vasoconstriction, which reduces the rate of absorption of local anaesthetics. Many studies have proved that dexamethasone when added as an adjuvant with local anaesthetics for TAP block, reduces VAS score, nausea, vomiting after the surgery, and usage of opioids [22,27]. It is assumed that corticosteroids produce their antiemetic activity by antagonism of prostaglandins.

Similar to naturally occurring glucocorticoids, methylprednisolone is a synthetic corticosteroid that acts systemically and has a broad variety of physiological effects. Due to methylprednisolone's anti-inflammatory and immunosuppressive properties, it is primarily utilised in clinical settings. Among the synthetic glucocorticoids with an intermediate-acting effect are methylprednisolone and its derivatives, such as methylprednisolone sodium and methylprednisolone acetate succinate. Their main uses are as anti-inflammatory or immunosuppressive medications. Methylprednisolone has minimal mineralocorticoid activity and five times the potency of hydrocortisone (cortisol) in terms of anti-inflammatory actions [28]. Similar to other corticosteroids, methylprednisolone also prevents the synthesis of cyclooxygenase (COX)-2, which is responsible for the production of prostaglandins in wounded tissue and the initiation of the inflammatory cascade [29]. Methylprednisolone suppresses immune responses mediated by cells, particularly those that rely on lymphocytes. Neutrophophilic leukocytosis, modest increases in monocytes, sharp decreases in circulating eosinophils, and minor decreases in lymphocytes are the outcomes of glucocorticoid therapy. Methylprednisolone and other glucocorticoids decrease leukocyte adhesion to vascular endothelium and cause subsequent intracellular adhesion. Many T cell functions are compromised by glucocorticoids, and at moderate-to-high doses, T cell death is induced whereas B cell function and antibody production are maintained [30].

Patients in the two groups in the study were comparable in terms of weight, age, gender, and duration of pain. As shown in Figure 2, mean VAS scores within the research groups were compared at various time points up to 12 weeks. The mean VAS among individuals in Group M was considerably lower than that of Group D in the fourth, sixth, and 12th weeks compared to baseline (zero weeks) values, which were similar in both groups. As a result, it was established that methylprednisolone, when used in conjunction with bupivacaine in TAP block, had superior analgesic efficacy than dexamethasone for at least 12 weeks. The quality of life score was significantly higher in patients of Group M in the fourth, sixth and 12th week compared to Group D, as shown in Figure 3.

The limitations of the study include the usage of a single dose of corticosteroid for all patients because of which the efficacy of various doses of the corticosteroid was not evaluated. 

## Conclusions

Compared to dexamethasone, the administration of methylprednisolone and bupivacaine enhances the quality and longevity of analgesia of USG-guided TAP block in patients with persistent abdominal pain. In the fourth, sixth, and 12th weeks, the mean VAS among the individuals in the methylprednisolone group was considerably lower than the mean VAS in the dexamethasone group. This makes it evident that methylprednisolone, when used in conjunction with bupivacaine in TAP block, has higher analgesic efficacy than dexamethasone for at least 12 weeks. In the fourth, sixth, and 12th weeks, patients in the methylprednisolone group had better quality-of-life scores than those in the dexamethasone group.
